# Autobiography of the Insane
*MS. Record of the History and Progress of Lunacy Cases.


**Published:** 1850-01-01

**Authors:** 


### Art V.—AUTOBIOGRAPHY OF THE INSANE.*

The human mind, by reflecting internally upon its own consciousness, is often enabled to analyze its faculties, and determine the laws by which they are governed, and by a similar process insane patients may themselves frequently account for, and throw light upon, certain states of mental aberration. The history given by them of the origin and development of their morbid impulses and delusions opens a curious field for psychological speculation, and one that has not hitherto been explored in this country. The existence of insanity, be it remembered, does not necessarily imply a complete overthrow and deprivation of all the reasoning faculties; the moral affections may be thoroughly perverted, and the propensities assume a wild and uncontrollable career, yet the intellectual faculties remain absolutely intact. The popular notion of insanity is that the unhappy lunatic is always in a state of bewilderment and incoherency; —hence, Mr. Charles Dickens introduces a madman's manuscript in his "Pickwick Papers," which, in accordance with this notion, is conceived in the following lofty and melodramatic strain:—"Yes—a madman—how that word would have struck to my heart many years ago—how it would have roused the terror that used to come upon me, sometimes sending the blood hissing and tingling through my veins till the cold dew of fear stood in large drops upon my skin, and my knees knocked together with fright. I like it now, though—it's a fine name.—Show me the monarch whose angry frown was ever feared like the glare of a madman's eye—whose cord and axe were ever so sure as a madman's gripe. Ho! ho! It's a grand thing to be mad—to be peeped at like a wild lion through the iron bars—to gnash one's teeth and howl, through the long still night, to the merry ring of the heavy chain—and to roll and twine among

* MS. Record of the History and Progress of Lunacy Cases.

the straw, transported with such brave music. Hurrah for the madhouse. Oh, it's a rare place!"

The late professor Charles Bell, in his "Anatomy of Expression," observes—

"To represent the prevailing character and physiognomy of a madman, the body should be strong and the muscles rigid and distinct, the skin bound, the features sharp, the eye sunk, the colour of a dark brown yellow tinctured with sallowness, without one spot of enlivening carnation, the hair sooty black, stiff, and bushy. Or, perhaps he might be represented, as in the "Faëry Queen," of a pale sickly yellow, with wiry hair.

"His burning eyen, whom bloody streakes did stain,
Stared full wide, and threw forth sparks of fire,
And more for rank despight than for great pain,
Shaked his long locks, coloured like copper wire,
And bit his tawny beard, to shew his raging ire."

You see him lying in his cell, regardless of everything, with a deathlike settled gloom upon his countenance. When I say it is a deathlike gloom, I mean a heaviness of the features without knitting of the brows or action of the muscles. If you watch him in his paroxysm you may see the blood working to his head, his face acquires a darker red, he becomes restless, then, rising from his couch, he paces his cell and tugs his chain; now his inflamed eye is fixed upon you, and his features lighten up into wildness and ferocity. The error into which the painter may naturally fall is to represent this expression by the swelling features of passion and the frowning eyebrow, but this would only give the idea of passion, not of madness. Or he mistakes melancholia for madness. The theory on which we are to proceed in attempting to convey this peculiar look of ferocity amidst the utter wreck of the intellect, I conceive to be, that the expression of mental energy should be avoided, and consequently all those muscles which indicate sentiment. I believe this to be true to nature, because I have observed (contrary to my expectation) that there was not that energy, that knitting of the brows, that indignant brooding and thoughtfulness in the face of madmen, which is generally imagined to characterize their expression, and which is so often given to them in painting. There is a vacancy in their laugh, and a want of meaning in their ferociousness. To learn the character of the countenance when devoid of human expression, and reduced to the state of brutality, we must have recourse to the lower animals, and study their looks of timidity, of watchfulness, of excitement, and of ferocity. If these expressions are transferred to the human face, I should conceive that they will irresistibly convey the idea of madness, vacancy of mind, and animal passion."

Here, then, we have the lunatic as described by the novelist, and the lunatic as depicted by the critical artist; but in each case it is the exaggerated representation of a maniacal condition which is always of short duration when it does occur, and which is very rarely met with in any asylum. The theory propounded by professor Charles Bell, that there exists in such maniacal cases a deficiency of mental energy, and that there is a want of meaning also in the ferociousness exhibited, is also incorrect, for, on the contrary, during these paroxysms of excitement, the mind is in the most vigorous state of exaltation, preternaturally energetic and self-willed, and so far from such ferociousness being unmeaning, it characterizes an irrevocable determination and a dangerous intensity of purpose which absorbs all other passions.

These descriptions of insanity apply to a state of maniacal furor only; but it is not right to take this as the common type of lunacy; for not unfrequently the lunatic, instead of being a repulsive personage exciting alarm and trepidation, proves to be a man of prepossessing appearance—fascinating manners—agreeable conversation—full of wit, learning, and anecdote. Such a person was a patient with whom we became acquainted. He had taken a fancy that his family had conspired together to poison him, and he would reason upon, and even struggle against, the delusion, which was nevertheless too strong for him to master. Poor fellow!—he died, and upon a *post mortem* examination, the valves of the heart were found ossified; and as physical sensations frequently give rise to erroneous mental impressions, it is probable that the idea of poisoning was suggested by the uneasiness which he felt whenever the stomach was distended with food. Everything he ate disagreed with him—the heart laboured to propel the blood through its ossified and constricted passages—the lungs became congested, and the breathing difficult—and in this state he was wont to exclaim, "The villains have been poisoning me again." Nevertheless, in his happier moments, a more charming companion we never met with; no one ever sat down in his society without being amused and interested, and no one went away without having derived some information from the extent of his reading and the great variety of his scientific and literary acquisitions. We followed him to the grave, and found afterwards, among his papers, an essay on the Existence of Good and Evil in the World, which is conceived in a lucid and elegant tone worthy even of Tillotson. Compare the manuscript of the imaginary madman, by Mr. Charles Dickens, with the following "Observations on Original Sin:"

"It is certain that considering the world in general, we remark a design, a order, an harmony, a perfection which announces the wisdom and power of its author; but considering it in detail, we discover a disorder so great, that we can scarce think at first but that an unjust or impuissant Being had formed the universe, or that a malevolent principle had troubled as much as he could the order established by a beneficent principle. To prove what I advance, it suffices to make some remarks on our species. Considering this species in general, or in each of its individuals in particular, man seems at first sight an accomplished creature : there is nothing better conceived, nothing more perfect than his external structure ; nothing better proportioned to his nature, or his use, than his limbs and sensitive faculties. Anatomy discovers in his body a thousand admirable parts, which by their connexion, relation, and destination, make a whole still more admirable.

"Considering man on the side of his mental faculties, he thinks, he generalizes his ideas, he judges of their relations and of their oppositions ; he determines, he acts ; he invests his ideas with terms or arbitrary signs ; he perfects his imagination and his memory ; he communicates his thoughts, he perfects all his faculties, he learns arts, sciences, and entire nature is submitted to him. But these perfections of man are amply counterbalanced by his defects. This body so accomplished is a prey to all evils : hunger, thirst, and other natural needs, an infinite number of diseases make war upon him without ceasing ; accidents of every kind surround him ; anything wounds, tears, bruises, or kills him ; the reciprocal and continued action of solids and fluids, the varied impressions of the elements sometimes destroy him suddenly, sometimes they alter him insensibly, and lead him to an unfortunate and insupportable old age, which is only terminated by death.

"Man is no better off on the side of his mind than on that of his body ; griefs, desires of every kind besiege him continually ; pride, avarice, envy, anger, render him hard, unjust, cruel, and proper to do mischief to his species, in doing good to himself. In a word, all concurs to show that the evil in itself far exceeds the good.

"Thus, as regards man, it is not badly divided from what we see ; for all the other species, all other individuals that exist in the universe, the entire universe, is the same ; everything that exists is a compound of good and evil, of order and disorder, of perfections and imperfections. This monstrous assemblage of things so opposite announces then, first, either two eternal, necessary, and independent principles, which produce all the good and evil that they can, or a sole principle, who is neither sovereignly good, nor sovereignly wise, nor sovereignly powerful.

"Such are the objections which might be urged against the present state of things ; but as nothing in the universe occurs from hazard, but from a First Cause (for against the existence of an evil principle the unity of design amidst all the confusion of good and evil, is conclusive) which is God. Evil in general, physical as well as moral, is a real existence, which nothing could not have created, for its power would then have equalled that of God, which is impossible. God could not have created it, for God is just and good, or what to us as individuals is moral and physical evil, is not so to him, or to the universe at large : another cause could not have produced it, for God has created everything that exists.

"Then we have to reconcile moral and physical evil with the goodness and justice of God.

"That those evils which good men suffer are not mere disorders, without tendency to the greater good of the whole, which will hereafter be set right, is evident; for God does not suffer disorders for no other end but to set them right afterwards. But they are necessary to the present state of the universe, and everything here is in order. Thus far God is good. When in the future mutations of this world, he shall render to every man according to his works, he will be just, and neither men nor animals have cause to complain. The murderer may himself be murdered—the man who has been cruel to his beast suffer at the hands of another the wrongs which he has heaped upon animals here. If physical disorders do not break the order of the universe, neither do moral. Both are much more intimately connected than is generally supposed.

'But errs not nature from this gracious end,
From burning suns when livid deaths descend,
When earthquakes swallow, or when tempests sweep
Towns to one grave, whole nations to the deep?
'No, ('tis replied,) the first Almighty cause
Acts not by partial, but by gen'ral laws;
Th' exceptions few; some change since all began,
And what created perfect?' Why, then, man?
If the great end be human happiness,
Then Nature deviates; and can man do less!
As much that end a constant cause requires
Of show'rs and sunshine, as of man's desires:
As much eternal springs and cloudless skies,
As men for ever temp'rate, calm, and wise.
If plagues and earthquakes break not Heav'n's design,
Why then a Borgia or a Cataline?
Who knows but He whose hand the lightning forms,
Who heaves old ocean, and who wings the storms,
Pours fierce ambition in a Cæsar's mind,
Or turns young Ammon loose to scourge mankind?
From pride, from pride, our very reas'ning springs;
Account for moral, as for natural things:
Why charge we Heav'n in those, in these acquit?
In both, to reason right is to submit.
Better for us, perhaps, it might appear,
Were there all harmony, all virtue here;
That never air or ocean felt the wind;
That never passion discomposed the mind.
But all subsists by elemental strife;
And passions are the elements of life.
The gen'ral Order since the whole began,
Is kept in Nature, and is kept in man.'"

With this extract from Pope's Essay on Man, which is so aptly applied, the fragment ends. But while he could reason thus clearly and ably, his moral apprehensions and affections were still perverted; and if the name of one particular member of his family were alluded to or mentioned, he would utter imprecations against him, and accompany them with the most bitter homicidal threats. Among his manuscripts was another document which contrasts somewhat strangely with the above; it is entitled "My Last Will and Testament," and clearly indicates the morbid state of his feelings. It is as follows:—

"In the name of God. Amen! This is the last Will and Testament of me, ——. I bequeath all my property, consisting of * * * in the Three per Cents., and about * * * in Messrs. —— Bank, and a security upon the Estate of ——, in the county of ——, which security is in my brother's desk, to Mr. ——, artist. To all my own family I bequeathe my curse for having administered and bribed other persons to administer poison to me by which I am reduced to a very weak state, and for having bribed two doctors to certify me insane, when I was not so, by which I have been confined for two years and six months without ever being insane. May Jehovah visit these wrongs upon them is the last prayer of ——."

We have intentionally contrasted these two documents—"look upon that picture and on this:" in the one case we have the clear-sighted and eloquent logician—albeit, insane—arguing a matter of profound theological interest: the mind is seen unclouded, reasoning closely and acutely upon the problem before it; in the other, we are startled by the evidence before us of the total perversion of the moral feelings and natural affections—the mind has now passed into another phase, and is seen obviously writhing within the shadow of its own cruel delusion. But peace be to the memory of our deceased friend! The grass has scarcely yet overgrown the mound above his grave in the cemetery of Kensal-green.

The next case we shall refer to is that of a patient who has himself, since his convalescence, furnished us with the following autobiographical sketch of his mental state during the accession of his malady. This gentleman (about thirty years of age) presented himself at an early hour in the morning at the gates of the asylum requesting admission. He was overcome with fatigue, having been wandering for several days and nights about the streets of the metropolis and its vicinity. He informed us, when we saw him, that he was a prophet of the Lord on his way to Jerusalem, and that the Holy Spirit had directed him hither to seek food and rest. He was obviously labouring under religious delusions, and after communicating with his friends, he was duly certified, and admitted. There is a popular fallacy abroad that the interior of Lunatic Asylums abounds with melancholy and revolting spectacles; the picture of Hogarth, the incoherency, ferociousness, and mental prostration described by Mr. Charles Dickens and Professor Charles Bell, are expected to be universally met with in all such establishments; but instead of this, under the present enlightened and humane system of treatment, the insane patients themselves form very frequently an agreeable and cheerful society, which proves mutually beneficial and even curative to one another; nay, strange as it may appear, certain forms of lunacy are actually cured by the society of lunatics. Thus the man who believes himself to be Julius Cæsar finds himself in the presence of another Julius Cæsar; he starts back, and challenges the right of his imperial rival to usurp the dignity of the Cæsars. He begins to argue the case with those around him,—then with himself,—when suddenly a light breaks in upon his brain: there cannot be two Julius Cæsars,—am I under a delusion? The doubt,—the distrust,—the self argumentation which follow, gradually lead to the restoration of his reason; he then discovers, and allows that he has been deceived, and awakes as from a dream. Such was the case with ——, who, after remaining some weeks in the Asylum without undergoing any particular medical treatment, became convalescent, and returned to his duties in the world. The history which he has himself given of the mental perturbation which he suffered upon the accession of his malady is psychologically curious. He was, it should be premised, a clerk, or steward in a gentleman's household.

"From July, 1847, to November of the same year, I was highly nervous, and experienced a considerable loss of strength and flesh; spoke sometimes so sharply to those around me as to startle them, and make them fear me. About this time (the beginning of the attack) I felt great anxiety for the eternal salvation of my employer. His brother was lying ill, and I begged that I might visit him, but my offer was refused; I, therefore, prayed earnestly for his recovery, and had the satisfaction of hearing next day that he was better. Strong hope, mingled with fear, now took possession of me. When at prayer something would pull at my back, blow in my face as if in derision, and, hovering round my mouth, try to snatch the words from my lips. At night, when in bed, I felt something press upon my chest, and awoke in great trepidation in the middle of the night, when I sometimes heard music at a distance. These impressions terrified me so much that I dreaded to lie down; then again, I was afraid of forfeiting God's confidence by committing some undefined sin that I could not resist. Therefore I felt a strong inclination to leave the house of my benefactor, which desire was increased by my imagining that the persons in it would fall into apostasy. Hence I had recourse to prayer with all my heart, with all my power; and while praying nearly fainted. It next occurred to me that my employer had become rich by unjust gains, and that he and his wife would be trodden down in the streets and trampled to death. One evening, while at prayer, I saw a circle descend slowly on my head, and afterwards told my wife that I was the anointed of the Lord, but she did not appear to understand my meaning. Felt that I was very ignorant of the Scriptures; but expected every day that the power of God would instruct me, and that I should be commanded to leave the house on a sudden: so I put all things in order for my departure. On the 9th of March I left; but I was greatly agitated, and wept frequently, being unable to restrain my feelings. About this period, I began to see objects like gnats floating before my eyes, and thought that they were wicked spirits watching me; however, I felt satisfied that I was anointed in a very high degree, and that my mission from the Holy Spirit was to walk incessantly about, and convert the people I met with. As I passed near to them, I believed the Holy Spirit transferred itself from me to them; so I selected the most crowded thoroughfares in the metropolis for the work of conversion, and extended my walks daily, sometimes even into the adjoining counties, and I thought the people often turned round, and looked at me as I passed, with great satisfaction, as if conscious of the blessing I had conferred on them. To see the crowds I had converted greatly encouraged me in my labours; and now, delighted with my office, I had special revelations. One night, while in bed, I saw the glory of the moon; it was like an horizontal pillar across the moon, which increased in size and radiance as it approached my bed-room window, and I now believed that I was to be a prince, and the high priest of our Saviour. Upon the approach of the morning I felt a burning flame around me, and conceived that it was the glory of God sanctifying me for the work I had to perform. My sensations frequently alarmed me; more than once I was afraid I should go mad, and then I alternately laughed and wept. One day, I heard my feet speaking to me, telling me that I should be a king and reign at Jerusalem; and I also heard other voices telling me that I was Dan, the Son of Jacob, and should have large possessions at Jerusalem. Thus, having left my home, I wandered over miles of ground, imagining that I was forbidden to sit down or stand still, and, after having walked the whole night, one morning I arrived in Sion Lane, and was, by one of the cottagers, conducted to the house, where I expected to find food and rest. The proprietor, I supposed, was a high churchman; and I expected all the inhabitants would come while I was asleep, and look at me, in order that they might be converted. During the first few weeks of my residence there, many strange fancies came across my brain; with my new companions, and the medical gentlemen, I conversed freely, and gradually became quite conscious that I had been under delusions which have happily passed away, and my mental health is now, I am grateful to believe, quite restored."

The physiological condition of the system in this case,—the disturbed state of the cerebral circulation, indicated by the muscæ volitantes and aural illusions—the visions so analogous to those which imposed on the imagination of Francis Xavier, Jacob Bæhme, Paracelsus, and a host of religious enthusiasts and mystics,—the consciousness and dread of impending insanity, and the abnormal sensations transferred even to the extremities of the body,—are all symptoms of derangement of the circulating and nervous systems, suggesting erroneous impressions to the mind. But monomania, of which this is not an example, inasmuch as the delusion was not restricted to a single idea, is not, as Fouville has shown, in its simple form, a disease of frequent occurrence. In the Annales Psychologique a case of homicidal mania is detailed, which has been transferred into one of the American journals of Insanity; it was officially verified, and supplies a very remarkable autobiographical account of a state of lunacy, which presents many curious and interesting features.

"I, the undersigned, William Calmeilles, health officer, residing in the principal town of the canton of Cazals (Lot), certify to all whom it may concern, that upon the requisition of the mayor of the commune of Marminiat, I have this day been to the village of Brunet, in the aforesaid commune of Marminiat, to decide upon the mental condition of a person named John Glenadel, a husbandman, dwelling in the said village of Brunet.

"I found Glenadel sitting upon his bed, having a cord around his neck, fastened by the other end to the head of the bed; his arms were also tied together at the wrist with another cord. In giving my report, I do not believe that it can be better made, than by recording the conversation which took place between Glenadel and myself, in the presence of his brother and sister-in-law.

"*Question.* Are you unwell?

"*Answer.* I am very well; my health is excellent.

"*Q.* What is your name?

"*A.* John Glenadel.

"*Q.* What is your age?

"*A.* I am forty-three; I was born in '96, see if this is not correct.

"*Q.* Is it by compulsion or by your own consent that you are bound in this manner?

"*A.* It is not only by my consent, but I demanded that it should be done.

"*Q.* Why is this?

"*A.* To restrain me from committing a crime of which I have the greatest horror, and which, in spite of myself, I am constantly impelled to execute.

"*Q*. What is this crime?

"*A*. I have one thought which constantly torments me, and which I cannot conquer; that I must kill my sister-in-law, and I should do it were I not restrained.

"*Q*. How long have you had this idea?

"*A*. About six or seven years.

"*Q*. Have you any cause of complaint against your sister-in-law?

"*A*. Not the least, Monsieur; it is only this one unfortunate idea which troubles me, and I feel that I must put it in execution.

"*Q*. Have you ever thought of killing any one besides your sister-in-law?

"*A*. I at first thought of killing my mother; this thought seized me when I was fifteen or sixteen years old, at the age of puberty, in 1812, as I well recollect. Since that time I have not passed one happy hour; I have been the most miserable of men.

"*Q*. Did you conquer this unfortunate idea?

"*A*. In 1822, I could no longer resist, I being at that time, twenty-five or six years of age, and to remove this unfortunate inclination, I joined the army in the capacity of a substitute. I was two years in Spain with my regiment, and then returned to France, but this fixed idea followed me everywhere; more than once I was tempted to desert to go and kill my mother. In 1826, they gave me an unlimited furlough, although it was unsolicited by me, and I returned to my father's house, my fatal idea returning with me. I passed four years with my mother, always having an almost irresistable inclination to kill her.

"*Q*. What did you do then?

"*A*. Then, monsieur, seeing that I should inevitably commit a crime which terrified me and filled me with horror, I, in 1830, rejoined the army, that I might not succumb to this temptation. I left for the second time my father's house, but my fixed idea again followed me, and at last I almost decided to desert, that I might go and kill my mother.

"*Q*. Did you have any cause of complaint against your mother?

"*A*. No, monsieur, I loved her very much; thus, before starting, I said to myself, 'Shall I kill that mother who has exercised so much care over me during my infancy, and who has loved me so well, although I have entertained this fatal thought against her? I will not do it, but I must kill some one.' It was then that the thought of killing my sister-in-law first occurred to me; I have a distinct recollection of this, I being at that time in Dax, and it was in the year 1832. It was then announced to me that my sister-in-law was dead, which was a mistake, it being another relative who had died. I then accepted of the furlough they had offered me, which I should by no means have done, had I known that my sister-in-law was still living. When I reached my home, and was informed that she was not dead, I experienced such a sinking and depression of spirits, that I became quite sick, and my idea resumed its course.

"*Q*. What instrument do you choose with which to kill your sister-in-law.

"Here Glenadel was much affected, his eyes were bathed in tears, and looking towards his sister-in-law, he replied—'That instrument which would inflict the least pain!' But however that may be, the time approaches, I perceive, when she must die, and this is as certain as that God lives.

"*Q.* Do you not dread to inflict so much misery and anguish upon your brother and your little nephews?

"*A.* The thought of this has troubled me somewhat, but I should receive the punishment due to my crime, and should neither see nor know anything of their affliction; the world would rid itself of a monster such as me, and I should cease to live; I should not expect after this to see a single hour of happiness.

"It here occurred to me that M. Grandsault, of Salviat, my companion and friend, who is at present in Paris, had told me, about a year before, of a young man who, some years previously, had come, accompanied by his mother, to consult him as to his own case, which presented many features very similar to those exhibited by Glenadel; as these cases are so very uncommon, I thought that, perhaps, this person and Glenadel might prove to be the same. I therefore asked him if it was he who had consulted my friend, and he replied in the affirmative.

"*Q.* What did M. Grandsault counsel you?

"*A.* He gave me most valuable advice, and he also bled me.

"*Q.* Did you experience any benefit from this bleeding?

"*A.* Not the least; my unfortunate idea pursued me with the same force.

"*Q.* I am about to make a report upon your mental condition, from which will be decided whether you shall be placed in a hospital where you may recover from your insanity.

"*A.* My recovery is impossible; but make your report as quick as possible—time presses. I can control myself but a little longer.

"*Q.* It must be that your parents have instilled into your mind correct moral principles, that they have set before you good examples, and that you yourself have possessed a virtuous mind, to have resisted for so long a time this terrible temptation. Here Glenadel was again much affected, he shed tears, and replied, 'You are correct in this, Monsieur; but this resistance is more painful than death; I know that I can resist but little longer, and I shall kill my sister-in-law unless I am restrained, as sure as there is a God.'

"Glenadel," said I to him, "before leaving you, let me ask of you one favour: resist still for some days longer, and you shall not see your sister-in-law for a long time, as we will so arrange matters that you can leave here, since you so much desire it."

"Monsieur, I thank you, and I will make arrangements to comply with your recommendation."

"I left the house, and as I was about to mount my horse, Glenadel called me back, and when I had approached near to him, he said to me: 'Tell these gentlemen that I beseech them to put me in some place from whence it will be impossible for me to escape, for I should make attempts to do so ; and were I to succeed in getting away, my sister-in-law would have to die, for I could not avoid killing her; tell these gentlemen that it is my own self who has said this to you. I assured him that I would do this; but as I saw that he was in a state of great excitement, I asked him if the cord which bound his arms was strong enough, and if he did not think that by a strong effort he could break it. He made an attempt, and then said, I fear that I might. But if I should procure for you something that would confine your arms still more securely, would you accept of it? With thanks, monsieur. Then I will ask the commander of the *gendarmes* to give me that with which he is accustomed to confine the arms of prisoners, and I will send it to you.—You will confer upon me a great favour.

"I purposed to make many visits to Glenadel so as to entirely satisfy myself as to his mental condition; but after the long and painful conversation which I held with him, after what my friend M. Grandsault had told me, after what has been said to me by the brother and sister-in-law of Glenadel, who are so much afflicted at the sad condition of their unfortunate brother, I became well convinced, without farther observation, that John Glenadel was affected with that form of insanity called monomania, characterzied in his case by an irresistible inclination to murder; the monomania with which Papavoine, and others, fortunately but a small number, were affected.

"Signed at Brunet, in the commune of Marminiat.

"May 21, 1839. CALMEILLES, Health Officer."

For twenty-six years was this unfortunate individual haunted by this single idea; his general health was good; and for twenty years he resisted its impulsive promptings, during the whole of which period he conducted himself rationally, and maintained the appearance of being in a perfectly sound state of mind.

In the same journal another case is recorded of a woman, thirty years of age, named Augusta Wilhelmina Strohm, who, never having presented any appearance of melancholy, and without any apparent motive, killed, with a blow of a hatchet, one of her friends whom she had invited to her house, and immediately afterwards gave herself up to a police officer. Marc, after having reported this case in detail, continues thus :—

"When quite young, Augusta Strohm was present at Dresden, at the execution of a person named Schaefe, sentenced to death for assassination. The preparations for the execution, the procession to the scaffold, all produced upon Augusta Strohm such an impression, that from this moment she regarded it as the great and most desirable object of her existence to be able to terminate her life in the same manner; that is to say, to be prepared for death in the same way, and to close her life in as exemplary a manner as she who was condemned. This thought never left her; but her moral principles for a long time struggled against it, until, about six months previous to the event we are about to mention. the execution of an assassin, named Kultafen, took place at Dresden, &c.

"This second execution, by the circumstances with which it was accompanied, again made a very strong impression upon the girl Strohm, and sufficed to arouse the former idea which she had retained, and to impel this girl to the commission of murder. Strohm was quite young when she was present at the execution, from which dates the origin of her fixed idea. It is therefore probable, that for at least fifteen years did this fixed idea persist without producing any other disturbance of the mind, the patient preserving all the appearances of reason, notwithstanding the mental struggles she endured."

The persistency of this single idea coexisting apparently with the perception of its criminality, and the resolution to resist it, would appear to militate against the unity of consciousness; but then it is to be considered that these, instead of being coexistent; may have been consecutive mental states; for we cannot conceive the mind occupied with two different and distinct impressions at the same identical instant. "*Omne ens est unum*:" and upon this principle Lord Brougham, in a case of appeal heard lately before the Privy Council, contended that the term "partial insanity," is improperly applied to these cases; because the mind being one and indivisible, we cannot correctly speak of its different faculties being diseased. But this appears to be a mere verbal criticism; for, as Lord Brougham allows, nothing can be more certain than the existence of mental disease of this description: and the difficulty at once vanishes, if, instead of regarding these as *coexisting*, we view them as *consecutive* states of mind, following each other with the rapidity which characterizes the succession of all mental phenomena. The recurrence of an insane idea at different and distant intervals during a long series of years, and the consequent return of the same insane impulse, cannot, upon any materialistic theory of insanity, be explained; because, supposing this diseased manifestation originated in some physical alteration in the structure or condition of the brain, the cause being purely organic and permanent, the effect would be always present.

Here we may remark, that there are two theories of insanity—the one materialistic, the other purely psychological; the one teaches that the disease originates from, and is dependent upon some physical change in the structure of the brain; the other, that the mind may itself be subject to aberration. Both of these theories may be considered, as each presents us with a different form of insanity.

The human mind derives its knowledge from two sources—the one is external or objective, the other internal or subjective. Impressions are conveyed to the mind, in the former case, by the senses; and when these are diseased, they may unquestionably become the channels of false impressions. This is not all. As the integrity of all the vital functions is so immediately dependent on the state of the circulation, any cause which retards or accelerates the flow of blood through the brain and nervous system, will disturb and variously affect the mind. Upon the accession of fever there is often a very remarkable degree of mental lucidity, occasioned, doubtlessly, by the increased flow of arterial blood through the cerebral substance; the perceptions become morbidly acute, and all the intellectual faculties preternaturally excited.

The physical cause of this state is here obvious. In the latter case, however, which regards the internal sources of our knowledge, as the mind subjectively, or within itself originates ideas which are independent of external impressions,—so may these, from internal causes be erroneous or deranged. The fountain of our rationality may itself give rise to wrong and inconsistent principles of action. Thus, when the sense of moral discernment, or the natural power of distinguishing between right and wrong, is obscured or perverted, a purely mental power is affected. The mind through the bodily senses may receive false impressions; but, independent of these it may, from the arrangement of its own internal constitution, form false judgments, and come to irrational and extravagant conclusions. If, indeed, we concede that the mind is a principle independent of the body, however intimately associated with it in all its organic relations, there can be no reason for denying that it may, from various moral and psychological causes, be subject to certain states of aberration; and although the term mental disease is applied to the mind in this state, we are not to suppose that such a condition is at all analogous to any disease of the body. A state of aberration does not imply any disintegration of the mental principle; it is only aberrant with respect to its relation with the external world.

We believe, indeed, that a diagnosis may clearly be established between the physical and psychological form of insanity—between that disease as it arises froms bodily causes, and is dependent on certain pathological states of the brain and nervous system; and the disease as it arises from and consists in a certain derangement in the constitution of mind itself. We have in the one instance, evidence of functional disturbance in various organs of the body—the pulse, the appetite, and the secretions generally are affected; in the other, the same functions remain intact, and the general health is undisturbed. In the one, medical treatment in the early stage of the disease is of great importance, and the prognosis—which, *ceteris paribus*, must always be dependent on age, temperament, and other circumstances—is generally favourable : in the other, the disease yields only to moral treatment—medicines are of little use, and may even be pernicious—and the prognosis is almost always unfavourable. This distinction we have not before seen drawn, but it is fully borne out, not only by the phenomena of the disease, which we have attentively observed, but by the result of post-mortem examinations; for it is well known, that in a vast number of cases of insanity, the most expert and acute pathologists have failed to detect any morbid appearances whatever in the brain. These were, in all probability, cases of simple mental aberration, not dependent on any change in the cerebral substance.

Hitherto philosophers have studied the constitution of the human mind by reflecting upon their own consciousness, and the analysis so conducted has laid the foundation of all the metaphysical systems of any importance that have yet been established; but if these views be correct, it becomes important to study the different phases of the mind in states of disease—hence, the account which patients give of their own thoughts and feelings, impulses and delusions; in other words, the autobiography of the insane may throw considerable light the science of Psychology.

---

